# Physical mechanisms of ESCRT-III–driven cell division

**DOI:** 10.1073/pnas.2107763119

**Published:** 2022-01-04

**Authors:** Lena Harker-Kirschneck, Anne E. Hafner, Tina Yao, Christian Vanhille-Campos, Xiuyun Jiang, Andre Pulschen, Fredrik Hurtig, Dawid Hryniuk, Siân Culley, Ricardo Henriques, Buzz Baum, Anđela Šarić

**Affiliations:** ^a^Department of Physics & Astronomy, University College London, London WC1E 6BT, United Kingdom;; ^b^Institute for the Physics of Living Systems, University College London, London WC1E 6BT, United Kingdom;; ^c^Medical Research Council Laboratory for Molecular Cell Biology, University College London, London WC1E 6BT, United Kingdom;; ^d^Medical Research Council Laboratory of Molecular Biology, University of Cambridge, Cambridge CB2 0QH, United Kingdom

**Keywords:** cell division, archaea, ESCRT-III, membrane simulations, soft matter

## Abstract

Living systems propagate by undergoing rounds of cell growth and division. Cell division is at heart a physical process that requires mechanical forces, usually exerted by assemblies of cytoskeletal polymers. Here we developed a physical model for the ESCRT-III–mediated division of archaeal cells, which despite their structural simplicity share machinery and evolutionary origins with eukaryotes. By comparing the dynamics of simulations with data collected from live cell imaging experiments, we propose that this branch of life uses a previously unidentified division mechanism. Active changes in the curvature of elastic cytoskeletal filaments can lead to filament perversions and supercoiling, to drive ring constriction and deform the overlying membrane. Abscission is then completed following filament disassembly. The model was also used to explore how different adenosine triphosphate (ATP)-driven processes that govern the way the structure of the filament is changed likely impact the robustness and symmetry of the resulting division. Comparisons between midcell constriction dynamics in simulations and experiments reveal a good agreement with the process when changes in curvature are implemented at random positions along the filament, supporting this as a possible mechanism of ESCRT-III–dependent division in this system. Beyond archaea, this study pinpoints a general mechanism of cytokinesis based on dynamic coupling between a coiling filament and the membrane.

## Significance

Cell division is an essential requirement for life. Division requires mechanical forces, often exerted by protein assemblies from the cell interior, that split a single cell into two. Using coarse-grained computer simulations and live cell imaging we define a distinct cell division mechanism—based on the forces generated by the supercoiling of an elastic filament as it disassembles. Our analysis suggests that such a mechanism could explain ESCRT-III–dependent division in *Sulfolobus* cells, based on the similarity of the dynamics of division obtained in simulations to those observed using live cell imaging. In this way our study furthers our understanding of the physical mechanisms used to reshape cells across evolution and identifies additional design principles for a minimal division machinery.

Cell division is one of the most fundamental requirements for the existence of life on Earth. During division the material from a single cell is divided into two separate daughter cells. This is an inherently physical process. Living cells have evolved multiple ways to apply mechanical forces for this purpose. In general, division is thought to be achieved by proteins that assemble into long polymeric filaments at the cytoplasmic side of the cell membrane. These filaments then undergo a series of energy-driven changes in their form and organization to deform the associated membrane and/or guide cell wall assembly. However, the physical mechanisms by which this type of nonequilibrium protein self-assembly produces the mechanical work needed to reshape and cut soft surfaces remain underexplored.

Although the mechanisms of division differ across the tree of life, recent data support the idea that eukaryotic cells likely arose from the symbiosis of an archaeal cell and an alphaproteobacterial cell, where the archaeal host gave rise to the eukaryotic cell body and the associated proteobacteria went on to become mitochondria (1–3). Because of this, many physical processes that control eukaryotic cell division are likely to have originated in archaea. In particular, ESCRT-III filaments, which drive cell division in a subgroup of archaea called TACK (thaumarchaeota, aigarchaeota, crenarchaeota and korachaeota) archaea, also catalyze the final step of cell division in many eukaryotes (4, 5).

**Fig. 1.**
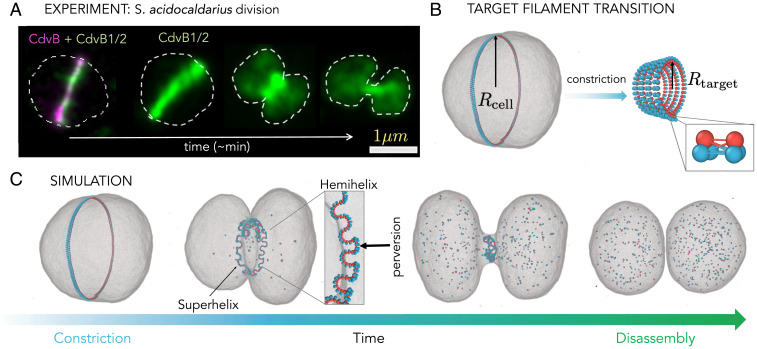
Computational model. (*A*) Division of an archaeon *S. acidocaldarius*. The cell membrane is indicated with a dashed line, the template filament (CdvB) is fluorescently labeled in magenta, while the constricting ESCRT-III filament (CdvB1) is labeled in green. (*B*) The initial ESCRT-III filament state (CdvB + CdvB1/2) is modeled as a single helical filament with a target radius equal to the cell radius $R_{\text{cell}}$. The filament is attached to the inside of the fluid vesicle that represents the archaeal cell. To constrict, upon CdvB degradation, the ESCRT-III filament (CdvB1/2) reduces its target radius to $R_{\text{target}}$, which results in a new target state of a tighter helix. The filament model itself consists of triplet subunits that are connected to each other via nine bonds whose lengths control the filament curvature (*Inset* and *SI Appendix*). (*C*) An example of a cell division simulation. The target radius of the filament is instantaneously decreased to 5% of the original cell radius. The filament is then disassembled from both ends at a rate $10^{2}\times v_{\text{dis}}=6.7/\tau$ (*τ* is the molecular dynamics unit of time). The filament first forms a superhelix that consists of multiple short helices of alternating chiralities (shown in the box). As the superhelix contracts and disassembles, it pulls the membrane into a tight neck, which spontaneously breaks (Movie S1).

Here we develop a physical model to study the dynamics of archaeal cell division by ESCRT-III filaments. In archaea, ESCRT-III proteins polymerize into at least two distinct filamentous rings that likely form a copolymer that is adsorbed on the cytoplasmic side of the cell membrane. The first filamenteous ring (called CdvB) serves as a template for the assembly of a contractile ring (made of CdvB1 and CdvB2 proteins) (Fig. 1*A*). Recently it has been shown that the contractile CdvB1/2 ring is free to exert forces to reshape the membrane only once the template CdvB ring has been removed (6). As cytokinesis proceeds, the contractile CdvB1/2 ESCRT-III filament is then disassembled.

Here we investigate different energy-driven protocols that can lead to the contraction and disassembly of an ESCRT-III ring in contact with a deformable cell. We quantify the rates, reliability, and symmetry of the resulting cell division processes. We then compare the dynamics of cell division predicted in simulations with those observed via live imaging of the archeon *Sulfolobus acidocaldarius*, the closest archaeal relative to eukaryotic cells that can be easily cultured in a laboratory. This comparison identifies a single regime of filament remodeling that matches the experimental data remarkably well.

Our simulations identify a physical mechanism for reshaping and splitting cells in which division is driven by the supercoiling of the filament. This differs from models of division described previously but, given the generality of our modeling approach, suggests a possible role for this process in cytoskeletal-induced membrane deformation events across biological systems. In this way, our analysis should help inform efforts to design synthetic nanomachinery that can drive the division of synthetic cells (7–9).

## Results

### A Minimal Model of Cell Division

In the model, the ESCRT-III filament (CdvB1/2) bound to the template (CdvB) is described as an elastic helical polymer made of two full turns whose radius of curvature matches the radius of the cell, $R_{\text{cell}}$ (Fig. 1*B*). The polymer is modeled via three-beaded monomers that are connected by harmonic bonds to each other (Fig. 1 *B*, *Inset*). The equilibrium lengths of the bonds determine the curvature of the polymer (10). The archaeal cell is described as a vesicle made of a coarse-grained, one-particle-thick membrane (11), in which a single particle corresponds to a cluster of ∼10-nm-wide lipid molecules. These particles interact via an anisotropic pair potential that drives the self-assembly of fluid membranes with expected physiological bending rigidity. The outer side of the filament (blue-colored beads in Fig. 1*B*) is adsorbed to the membrane via a generic Lennard-Jones attractive potential, while the inner side of the filament (red beads) interacts with the membrane only via volume exclusion. The system is evolved using molecular dynamics simulation with a Langevin thermostat (see *Materials and Methods* and *SI Appendix*, section 1 for more details). Such a model captures thermal fluctuations that are needed for the relaxation of the filament, the dynamic filament–membrane attachment and detachment, and the spontaneous membrane neck scission.

The removal of the template CdvB filament and subsequent constriction of the ESCRT-III filament (CdvB1/2) are modeled by shortening the equilibrium lengths of the bonds between the filament’s subunits, which increases the filament curvature, from a large radius of curvature $R_{\text{cell}}$ to a small radius $R_{\text{target}}$. Fig. 1*B* shows the filament in its initial and target geometrical states. This change in target filament radius is expected to transform the filament from a wide ring into a tight helix that will drag the membrane with it. To test this idea, we instantaneously changed the target radius of the filament to 5% of the cell radius and followed the system as it evolved. Since filament disassembly is needed for scission (6), as soon as the new equilibrium bond lengths were imposed, the disassembly was initiated by severing bonds between filament monomers sequentially from both ends of the helix at a specified rate $v_{\text{dis}}$.

Interestingly, instead of forming a single tight helix, which is its target shape, upon constriction the filament rapidly transforms itself into a collection of short tight helices with alternating chirality separated by kinks, termed perversions (Fig. 1*C*). This structure is called a hemihelix and it presents a local energy minimum in which the system gets trapped (12–14). This shape arises as the result of nonhomogeneous stresses in the filament, such that the outer and inner portions of the filament are strained to different extents as the filament undergoes a change in curvature. Hemihelices, first documented by Darwin in plant tendrils (15), are ubiquitous in nature and have been reported to form in the development of the gut tube (16), in synthetic elastomers (17, 18), and can be seen in everyday life when a wrapping ribbon is induced to curl using scissors (19). Recently, this shape has also been imaged in nanoscale biopolymers such as mitotic chromosomes in vivo (20) and, importantly for this analysis, hemihelices are visible in many of the published in vitro images of ESCRT-III and FtsZ filaments (see examples in *SI Appendix*, Fig. S13 or figure 1b in ref. 21, figure 5 g–i in ref. 22, or figure 6 in ref. 23).

When attached to the cell, the hemihelix itself acquires a superhelical shape to follow the cell curvature (Fig. 1*C*). This effectively shortens the filament length and thereby pulls the membrane with it. Over time, the filament further shortens as it disassembles, until it becomes a single helix with a curvature $1/R_{\text{target}}$ enveloped by a tight membrane neck. As this helix disassembles, the membrane neck spontaneously breaks, giving rise to two separate cells (Movie S1). To the best of our knowledge, this mechanism of constriction—coiling of a coil—has not previously been considered as playing a potential role in cytoskeletal filament-based membrane deformation in cells.

### Characterizing the Reliability and Symmetry of Cell Division

**Fig. 2.**
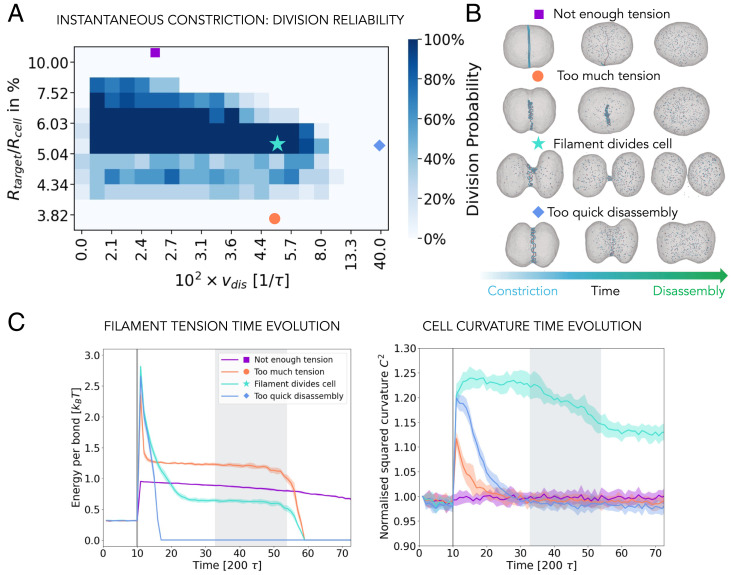
Reliability of division. (*A*) The influence of the filament radius reduction, $R_{\text{target}}/R_{\text{cell}}$, and the rate of the filament disassembly, $v_{\text{dis}}$, on the probability of cell division. The color of each square represents the percentage of successful divisions out of 10 simulations performed with different random seeds. (*B*) Snapshots of the representative simulations from *A*, as indicated by the respective symbols. (*C*) Time evolution of the average filament tension (*Left*) and average membrane curvature (*Right*) for the four representative points from *A*, as indicated by the respective labels. The filament disassembly starts at the vertical gray line in all the cases, and the shaded gray region marks the time range during which successful divisions occur (turquoise curve and star symbol). All the curves show an average over 10 simulations.

We next carried out a series of functional tests to determine how reliable this process is as a division mechanism. Fig. 2*A* shows the dependence of the probability of cell division on the amount of the filament curvature change, $R_{\text{target}}/R_{\text{cell}}$, and the rate of the filament disassembly, $v_{\text{dis}}$. We see that division occurs only if both the filament curvature change and its disassembly rate are in the right regime. When the change in the filament curvature is insufficient, the resulting large hemihelical loops barely shorten the filament and the filament does not have enough tension to constrict the membrane to a width sufficiently narrow to induce fission. As the filament disassembles in this case, the cell inflates back to its original size (square labels in Fig. 2 *A* and *B*). In the opposite regime, if the reduction in the filament curvature is too large, the large tension stored in the filament drives its detachment from the membrane before a substantial deformation of the membrane can occur (circle labels in Fig. 2 *A* and *B*). The time evolution of the filament tension and the tension transferred into the membrane curvature (24) is shown in Fig. 2*C* for each case.

In the intermediate regime, the filament fulfils two conditions that allow it to enable division: It has enough tension that it can form a sufficiently tight constriction and it disassembles at a slow enough rate to enable reaching of the tight neck (star symbols in Fig. 2 *A–C*). However, if the rate of disassembly is too high, the filament disassembles before a membrane bottleneck can be formed that is sufficiently narrow to enable scission, leading to division failure (diamond in Fig. 2 *A–C*). On the other hand, in the absence of filament disassembly ($v_{\text{dis}}=0$), the filament frequently blocks the membrane neck, preventing efficient cell division, even though there are instances in which the membrane neck can still break, leaving the daughter cells connected by the filament.

We also wanted to determine how the mechanism of division influences division symmetry—since this is a parameter that we are able to measure in experiments. We therefore test how evenly the disassembled filament subunits are partitioned between the daughter cells for different curvature changes and disassembly rates, in the region of phase space in which division occurs. This evenness is quantified via parameter $E=2N_{\text{small}}/N_{\text{total}}$, where $N_{\text{small}}$ is the number of filament subunits in the less-filled daughter cell and $N_{\text{total}}$ is the total number of filament subunits.

**Fig. 3.**
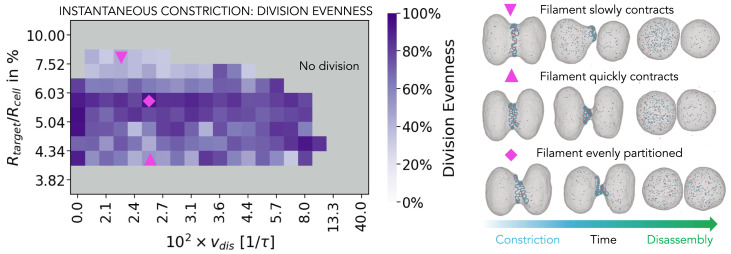
Symmetry of the division. (*Left*) The influence of the amount of the instantaneous target radius reduction and the rate of the filament disassembly on the partitioning of ESCRT-III proteins between the daughter cells. The color of each square represents average value of 10 simulations performed with different seeds. (*Right*) Representative simulation snapshots.

As shown in Fig. 3, for small filament curvature reductions, which are at the very boundary of the phase space in which division occurs (down-pointing triangle label), the filament does not constrict much and the division occurs because the membrane wraps over the exposed membrane attraction sites generated via local perversions. As a result, following division, one cell contains substantially more of the filament content than the other. This mechanism yields a successful division in only ∼70% of cases (Fig. 2*A*). A similar effect is seen for protocols that include a strong radius reduction (up-pointing triangle label in Fig. 3). Here filament supercoils are generated very quickly so that the membrane wraps over one side of the coiled filament and the filament is inherited by just one of the two daughter cells. The result is again a less reliable and more uneven division. In the middle of the dividing region (star label in Fig. 2 and diamond label in Fig. 3), the division machinery is evenly distributed between the daughter cells. In this region of the phase diagram division occurs with ∼100% probability.

### Dynamical Curvature Change Protocols

**Fig. 4.**
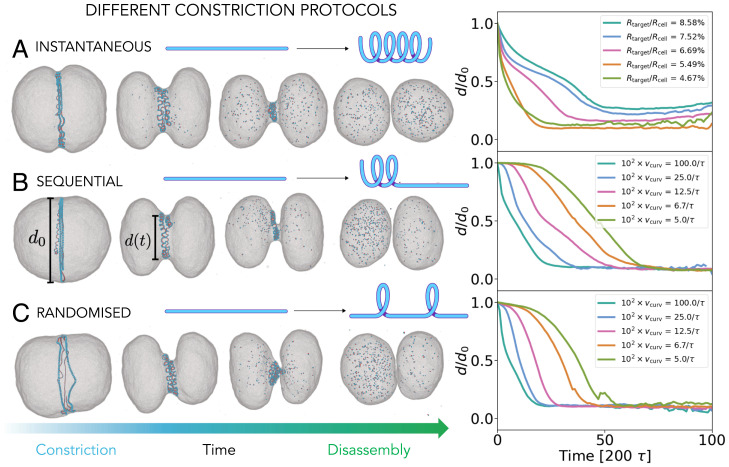
Different protocols for the curvature change. (*A*) Instantaneous: The target filament radius changes globally throughout the filament to $R_{\text{target}}/R_{\text{cell}}$, as indicated in the key, within a single time step. (*B*) Sequential: The filament curvature change starts at one end of the filament and propagates at a rate $v_{\text{curv}}$ to the other end, here shown for $R_{\text{target}}/R_{\text{cell}}=5.5\%$. (*C*) Randomized: Random bonds along the filament constrict at a rate $v_{\text{curv}}$, here shown for $R_{\text{target}}/R_{\text{cell}}=5.5\%$. In all the protocols, once the entire filament has transitioned, it is disassembled from both ends at a rate $v_{\text{dis}}$. The value of $v_{\text{dis}}$ does not influence the division curves (*SI Appendix*, Fig. S4). *A–C*, *Right* compare the normalized diameter of the cell at the midzone as a function of time. We selected only simulations that led to division and averaged over simulation seeds and different disassembly rates.

The choice to induce instantaneous changes in filament curvature (Fig. 4*A*) was inspired by experimental observations in *S. acidocaldarius*, where the template CdvB ring was found to be rapidly degraded, which was then assumed to trigger rapid curvature change of the constricting ring, akin to the release of a loaded spring (6). However, the local constriction time is nonzero and is likely to be set by the local presence of an enzyme Vps4 ATPase, which is needed for division in *Sulfolobus*, as well as for force production by ESCRT-III filaments in eukaryotes (25, 26). Pfitzner et al. (26) recently found that Vps4 modifies the composition of ESCRT-III copolymers in a stepwise manner, thereby ordering the changes in overall filament geometry to drive membrane remodeling. This led us to investigate how different protocols of noninstantaneous dynamical filament constriction influence the outcome of the division process.

We tested two additional protocols: 1) Filament curvature is changed in a sequential manner starting from one end of the filament in a process that spreads at a rate $v_{\text{curv}}$ (Fig. 4*B*), and 2) filament curvature is changed at random points along the filament at a rate $v_{\text{curv}}$ (Fig. 4*C*). In all cases the total change in filament curvature was kept constant (see *SI Appendix*, Fig. S5 for the exploration of different amounts of curvature change) and filament disassembly was initiated after the new target curvature had been imposed throughout the whole filament. To compare the effects of these different mechanisms of filament constriction for each of the different protocols we tracked changes in cell diameter over time.

As can be seen in Fig. 4*A*, *Right*, following an instantaneous curvature change the cell diameter sharply decreases and then slowly plateaus before the division occurs—as expected following the release of energy stored in a tensed polymeric spring. The smaller the target radius of the filament, the more energy is stored in the filament, and the faster the cell diameter reaches its equilibrium value. This behavior is in good agreement with the dynamic data for cytokinesis in animal cells reported by Turlier et al. (27).

The sequential and randomized protocols (Fig. 4 *B* and *C*) follow the same qualitative curve only if the curvature change is very fast, approaching the instantaneous limit. However, if the curvature change is implemented more slowly, something that is expected to mimic conditions in cells, the time evolution of the cell diameter follows a concave curve, where the furrow formation is significantly delayed, after which it follows an almost linear decrease before division occurs. The slower the constriction rate is, the longer the delay in the furrow formation, which indicates that a certain critical portion of the filament has to be constricted to significantly deform the membrane. This delayed onset is less pronounced for the randomized protocol, since the filament is transformed at random points. As a result, the tension is distributed evenly across the midcell diameter ring, leading to constriction that is faster than that seen when using the sequential protocol.

**Fig. 5.**
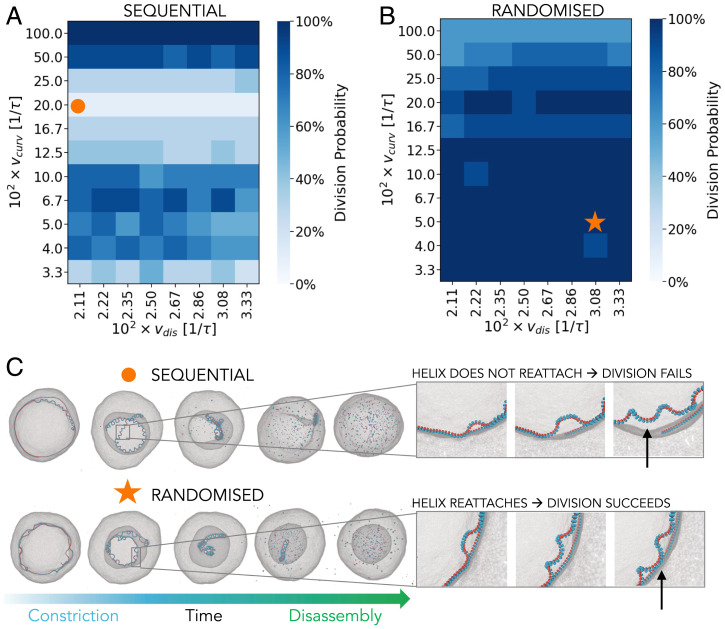
Reliability of division for noninstantaneous curvature change protocols. (*A* and *B*) The influence of the filament constriction rate, $v_{\text{curv}}$, and the filament disassembly rate, $v_{\text{dis}}$, on the probability of cell division for the sequential (*A*) and randomized (*B*) protocols. The color of each square represents the amount of successful divisions out of 10 simulations performed with different seeds. (*C*) The representative examples of a successful and an unsuccessful division. If the local helices fail to reattach to the membrane when forming (indicated by an arrow in *Upper panel*), division fails. If the helices manage to reattach to the membrane (arrow in the *Lower panel*), division succeeds. All simulations are performed using $R_{\text{target}}/R_{\text{cell}}=5.5\%$, which divides cells with 100% reliability for the instantaneous protocol (Fig. 2).

These observations can be rationalized by looking at the changes in filament geometry, which depends both on the protocol and on the rate of curvature change (*SI Appendix*, section 2). The faster the curvature change is, the more perversions the hemihelical filament contains (*SI Appendix*, Fig. S2), in agreement with previous studies of hemihelix formation in elastic materials (19). Importantly, the filament coiling in response to implementation of the randomized protocol contains more perversions (*SI Appendix*, Fig. S2) and as a consequence has a smaller filament radius of gyration (*SI Appendix*, Fig. S3*A*) and higher filament tension (*SI Appendix*, Fig. S3*B*) and therefore drives a greater membrane deformation (*SI Appendix*, Fig. S3*C*) than the filament coiled via the sequential protocol. This is because it is energetically costly to position perversions close to one another (14); hence, the clustered perversions formed in the sequential protocol are more likely to relax than in the case where they are randomly positioned throughout the polymer. Defect relaxation requires the filament to detach from the membrane and can lead to division failure (Fig. 5).

To further explore the differences between the sequential and random constriction protocols, we characterized the extent to which a robust cell division depends on the rate of curvature change and the rate of filament disassembly, fixing the amount of curvature change to $R_{\text{target}}/R_{\text{cell}}=5.5\%$ (Fig. 5 *A* and *B*). For the case of sequential curvature change, the cell divides reliably (Fig. 5*A*) and evenly (*SI Appendix*, Fig. S6*A*) only for very fast curvature changes, which approach instantaneous constriction. In that regime the new curvature and associated tension spread quickly across the filament length, pulling the membrane with it (Movie S2). However, if the rate of curvature change is decreased, the system enters a regime in which almost none of the cells divide. In this regime the uneven distribution of tension along the filament length comes into play as local perversions in the filament have time to detach from the membrane and to equilibrate while the rest of the filament is still transitioning (Fig. 5*C* and Movie S3). If the rate of curvature change is further decreased, division becomes more likely again, because the detached portion of the filament has time to reattach and pull the membrane with it.

For the randomized curvature change protocol (Fig. 5*B* and Movie S4) the curvature and the associated tension are distributed more evenly along the filament, independently of the rate, enabling more effective filament constriction (*SI Appendix*, Fig. S3) and resulting in a more reliable (Fig. 5*B*) and even (*SI Appendix*, Fig. S6*B*) division overall. The randomized protocol is also less sensitive to the choice of the filament’s target radius (*SI Appendix*, Fig. S5). From an energy perspective, we found that both curving protocols dissipate a comparable fraction of the energy that was invested into the curvature change (*SI Appendix*, section 7 and Fig. S8). However, in the radnomized protocol, the nondissipated portion of energy is used more productively, such that more of it is converted into the energy required to deform the membrane. Ultimately, our model predicts that cells divide more successfully and evenly if the constricting filament changes its curvature in a randomized fashion, rather than sequentially from one filament end.

### Comparison between Simulation and Experimental Data

**Fig. 6.**
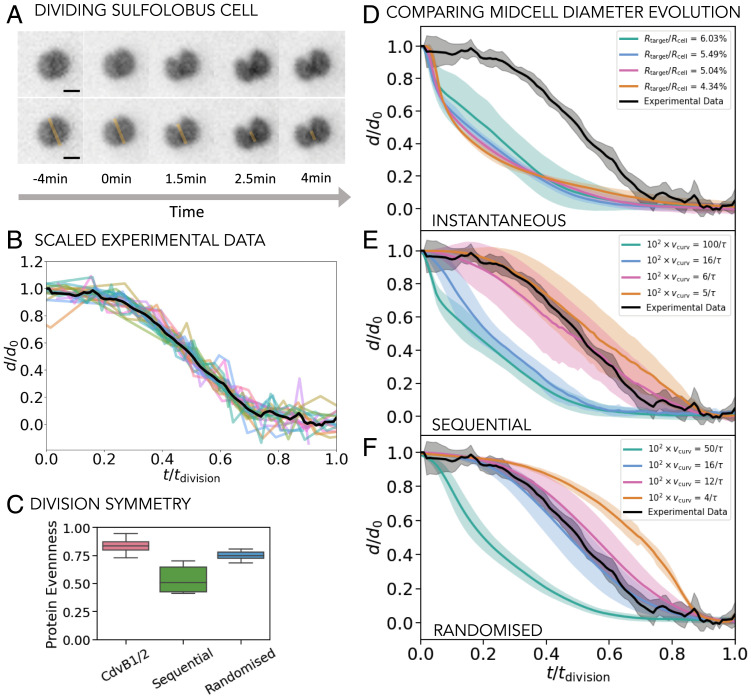
Comparison with live cell experiment. (*A*) Time sequence of a dividing *S. acidocaldarius* cell. *Bottom* row shows the division axis along which we measure the membrane intensity profile to determine the time evolution of the midcell (furrow) diameter. (Scale bar, 1 µm.) (*B*) The evolution of the midcell diameter in time. The midcell diameter *d* is normalized by the initial cell diameter *d*_0_, while the time is normalized by the total division time *t_division_*. Colored curves show the normalized measurements for individual cells (*N* = 23), and the black line shows the mean of all the experimentally measured curves. (*C*) The average of the partitioning of the constricting filament proteins in the daughter cells in experiments (CdvB1/2) and in simulations for noninstantaneous curvature change protocols that match the experimental curves for midcell evolution in time ($10^{2}v_{\text{curv}}=5/\tau$ for the sequential and $10^{2}v_{\text{curv}}=16/\tau$ for the randomized protocol). (*D*–*F*) The normalized cell diameter evolution curves collected in simulations for different protocols (*D*, instantaneous; *E*, sequential; *F*, randomized) and compared to the averaged experimental data (black curve). For the instantaneous protocol different amounts of filament constriction $R_{\text{target}}/R_{\text{cell}}$ are investigated. For the sequential and randomized protocols $R_{\text{target}}/R_{\text{cell}}=5.5$% and the constriction rate $v_{\text{curv}}$ is varied, as shown. The disassembly rate does not influence the curves. All the simulation curves are averaged over at least 10 repetitions and the shading shows 1 SD.

To identify which of the described constriction protocols best describes the ESCRT-III–driven archaeal cell division, we compared the time evolution of the midcell diameter collected in simulations with similar measurements taken from live dividing *S. acidocaldarius* cells. To do so, we analyzed cells imaged live using a membrane dye from Pulschen et al. (28), as shown in Fig. 6*A*. This setup allowed us to measure the intensity profile of the membrane along the division axis over time and hence extract the midcell (furrow) diameter evolution (Fig. 6*A* and *SI Appendix*, Figs. S11 and S12). Since cells can have varying initial diameters and can take varying lengths of time to divide, we rescaled the midcell diameter with the initial cell diameter and the time axis with the total division time for each cell. We then aligned all the curves at the point of 50% of the cell diameter decrease. Satisfyingly, all rescaled cells appear to follow the same general trajectory as they divide, enabling us to average the data to generate a single experimental curve (Fig. 6*B*; see *SI Appendix*, sections 10 and 11 for details). We then rescaled our simulations the same way to allow a direct comparison between model and experiment.

As can be seen in Fig. 6*D*, in simulations based on instantaneous filament constriction the behavior of the furrow diameter in time has a very different qualitative shape from the experimental curve, irrespective of the amount of curvature change in the filament. We considered the possibility that this discrepancy arose because the simulated cells are effectively empty on the inside and do not resist the fast initial cell constriction when the target filament geometry is suddenly changed. To determine whether bulk cytoplasm mass is likely to contribute in this way, we repeated the instantaneous constriction protocol measurements with cells in which the cytoplasm was modeled using volume-excluded particles. As *SI Appendix*, Fig. S9 shows, under these conditions the constriction curves did not change significantly, ruling this out as a contributing factor. Thus, the dynamics of the process appear to be governed by filament tension alone.

By contrast, the relatively symmetric initial and final plateaus seen for slower, noninstantaneous filament curvature change protocols resemble the membrane deformation trajectories seen in experiments. The randomized protocol shows somewhat better agreement with the experimental curves and, for a range of simulation parameters, the normalized simulation and experimental curves match remarkably well without any fitting (blue and pink curves in Fig. 6*F*). This randomized protocol also yielded a reliable division (Fig. 5*B*) and led to the symmetric partitioning of the division apparatus between the two daughter cells (*SI Appendix*, Fig. S6*B*).

Further, we measured the evenness of filament protein (CdvB1/2) partitioning in daughter cells in experiments and compared this with our simulations, as seen in Fig. 6*C*. The simulated data here are for the two best-matching curves of Fig. 6 *E* and *F* ($10^{2}v_{\text{curv}}=5/\tau$ for the sequential and $10^{2}v_{\text{curv}}=16/\tau$ for the randomized protocol). In the experiments, the CdvB1/2 filament proteins are divided fairly evenly between the daughter cells (Fig. 6*C*), in agreement with the behavior found in simulations with the randomized protocol. Taken together, these results suggest that a constriction protocol in which the filament curvature is noninstantaneously changed at random points throughout the filament length faithfully reproduces cell division as seen in *Sulfolobus*.

## Discussion

Here we develop a computational model for the dynamics of archaeal cell division via ESCRT-III filaments to study the physical mechanisms that underlie the division process. In doing so, we identify a cell division mechanism that is driven by the supercoiling of a disassembling elastic filament. Our mesoscale model allows for a direct connection between the geometrical and mechanical properties of the filament and the resulting experimentally observable cellular behavior. This model can now be used to test hypothetical division mechanisms that cannot yet be directly addressed in experiments and to make predictions about what one expects to see once the necessary tools become available.

One of the outstanding questions in the cytoskeletal and cell division fields has long been whether cytoskeletal elements like ESCRT-III are able to drive the entire process of cytokinesis, i.e., whether a single mechanism can be used to drive a membrane tube with a micrometer-sized diameter (the approximate size of a dividing *Sulfolobus* cell and a human midbody) to contract to a diameter sufficiently small to allow for spontaneous scission. Our model shows that this is possible and suggests a potential mechanism for crossing the two important length scales involved: the global curvature that arises due to the superhelical arrangement of a coiled filament and the local curvature of the individual coils. This supercoiling shortens the filament and guides the initial cell constriction, while the membrane wrapping around the individual tight coils creates a narrow neck that snaps after the filament has completely disassembled. While perversions like those predicted under this model of constriction can be clearly seen in images of ESCRT-III filaments reported in in vitro studies (*SI Appendix*, Fig. S13), as well as for FtsZ filaments (23), no one has yet assigned them a potential function. Based on our analysis, if this is how Sulfolobus cells divide, it should be possible to see ESCRT-III filament supercoils in dividing *Sulfolobus* cells via cryogenic electron microscopy experiments in the future. It will also be fascinating to determine whether similar structures are seen for other membrane-associated filaments.

Our analysis also suggests the time evolution of the furrow diameter of dividing *Sulfolobus* cells is best explained by a random model of filament curvature changes along the filament, which we predict would be due to stochastic binding of the Vps4 ATPase to the CdvB/B1/B2 copolymer. These random local changes in filament geometry serve to spread tension, facilitating a reliable and symmetric division. It can be imagined that such a mechanism is also the easiest one for cells to implement, as it does not rely on starting at a certain point within the filament.

Our simulations predicted that changing the filament’s preferred curvature away from the ideal region causes a more asymmetric division (see Fig. 3 for instantaneous and *SI Appendix*, Fig. S5 for sequential and randomized protocols). In experiments, asymmetric divisions occur when the CdvB2 protein is absent from the composite polymer filament. This may change the preferred filament curvature and has been found to cause filament detachment and slippage, followed by asymmetric division (28). It has also been suggested that the loss of CdvB1 might reduce filament tension, causing the observed occasional division failure and reduced midzone constriction rates (28). If this were the case, our simulations show how a decrease in tension in the filament can render division unreliable (*SI Appendix*, Fig. S7).

Thinking more broadly about division, we note that the important dynamic shape parameter used in this analysis—midzone membrane constriction in time—which proved so useful in comparing simulations and experiments, has been subjected to a thorough analysis in only a very small number of studies (27) even in more standard model systems (29, 30). Our analysis shows how extending this set of dynamic data will be very useful in gaining a mechanistic understanding of division across systems going forward, helping to reveal whether or not there are likely to be general rules that reflect common mechanistic principles.

Thus, we believe our model can be used as a starting point for the study of more complex division mechanisms that evolved from this minimalistic archaeal division, such as severing of the midbody in the last step of eukaryotic division, and may help us to understand division of soft compartments by nanoscale filaments in general. Finally, our model presents an excellent playground for testing specific nonequilibrium protocols behind bio-inspired nanomachines and their connection to the resulting nanomachine function. We therefore hope that our near-minimal model for cell division in this relatively simple archaeal cell system will also be of interest to those aiming to achieve the scission of synthetic cells, using reconstituted ESCRT-III filaments in vesicles.

## Materials and Methods

### Simulation

The simulation setup—an elastic self-avoiding helical filament that is coupled to the inside of a spherical membrane, representing the archaeal cell—can be seen in Fig. 1*B*. The filament helix has two full turns that consist of *N_total_* = 480 subunits. The ratio between the helix width and diameter (6.5σ/105σ≈0.06) corresponds roughly to the experimentally measured ratio (0.1 μm/1.25 μm=0.08) (6).

We model the filament using the ESCRT-III model we developed in Harker-Kirschneck et al. (10). It is built of three-beaded rigid subunits that are bonded to each other via nine strong springs with spring constant $K=600\;{\text{k}}_{\text{B}}T$ (Fig. 1 *B*, *Inset*), where ${\text{k}}_{\text{B}}$ is Boltzmann’s constant and *T* is temperature. The equilibrium lengths of the individual springs determine the target filament geometry (*SI Appendix*, section 1). The cell membrane is simulated by a coarse-grained, one-particle-thick model [developed by Yuan et al. (11)], in which a single particle corresponds to a lipid patch of ∼10 nm. These particles interact via an anisotropic pair potential that drives the self-assembly of fluid membranes with bending rigidity of $15{\text{k}}_{\text{B}}T$. The blue beads of the filament are attracted to the membrane via a Lennard-Jones potential, while the red beads interact with the membrane only via volume exclusion. A more detailed description of the simulation setup and all the interactions can be found in *SI Appendix*, section 1. We also repeated our main results using a triangulated membrane model within a Monte Carlo scheme (31), to show that our results are robust against a different membrane model (*SI Appendix*, section 9).

To change the filament curvature, the rest length of the bonds between the subunits is adjusted to the target curvature. In the case of noninstantaneous curvature change, the rate of the curvature change is determined by the number of bonds that are shortened per simulation timestep (0.01*τ*); i.e., if we constrict the bonds between two subunits every *n*th time step, the rate is $v_{\text{curv}}=1/(n(0.01\tau ))$. To simulate filament disassembly, every *m*th simulation time step one subunit is removed from each end of the filament, making the disassembly rate $v_{\text{dis}}=2/(m(0.01\tau ))$.

To measure the diameter *d* of the membrane at the cell midzone as a function of time, we collect all membrane particles that are located in a cuboid that is fixed at the center of the simulation box and has a width of 1σ (the size of one membrane bead). The collected coordinates are then projected onto a plane and the resulting data are fitted by a circle using the Taubin method (32).

This membrane model has not been developed to capture the membrane hydrodynamics (33); however, the timescales we describe (on the orders of minutes) are much longer than those of the expected relaxation of hydrodynamics effects in the cell membrane or in the cytoplasm (*SI Appendix*, section 8). This coarse-grained model is also not appropriate for capturing fine geometries of the membrane neck scission, where molecular details of the bilayer might become important. We nevertheless base all our comparison with experiment, and our mechanistic conclusions, on the first half of the cell division process, when the cell diameter constricts up to 50%, well before the tight neck geometries.

### Experiment

*Sulfolobus* cells were grown in standard media and imaged using Nile Red for membrane staining, as described by Pulschen et al. (28). This staining allowed us to measure the intensity profile of the membrane along the division axis over time (*SI Appendix*, Figs. S11 and S12). The full width at half maximum (FWHM) of this profile was used as a measure of the midcell diameter of the dividing cells, a parameter that we can compare well to our simulation results (for details see *SI Appendix*, section 10). *SI Appendix*, section 11 describes the rescaling procedure of the data. It is important to add that for the last part of the midcell evolution curve the experimental uncertainty in measurement becomes larger due to the resolution limit; hence, in all our results we focus on and discuss only the first half of the division curves.

## Supplementary Material

Supplementary File

Supplementary File

Supplementary File

Supplementary File

Supplementary File

## Data Availability

The simulation input files and codes are freely available at https://github.com/cvanhille/ESCRTIII_CD_ex and at University College London Research Data Repository: https://doi.org/10.5522/04/16601753.

## References

[1] D. A. Baum, B. Baum, An inside-out origin for the eukaryotic cell. BMC Biol. 12, 76 (2014). 25350791 10.1186/s12915-014-0076-2 PMC4210606

[2] A. Spang ., Complex archaea that bridge the gap between prokaryotes and eukaryotes. Nature 521, 173–179 (2015). 25945739 10.1038/nature14447 PMC4444528

[3] B. Baum, D. A. Baum, The merger that made us. BMC Biol. 18, 72 (2020). 32580772 10.1186/s12915-020-00806-3 PMC7315558

[4] A. C. Lindås, E. A. Karlsson, M. T. Lindgren, T. J. Ettema, R. Bernander, A unique cell division machinery in the Archaea. Proc. Natl. Acad. Sci. U.S.A. 105, 18942–18946 (2008). 18987308 10.1073/pnas.0809467105 PMC2596248

[5] M. J. Dobro ., Electron cryotomography of ESCRT assemblies and dividing Sulfolobus cells suggests that spiraling filaments are involved in membrane scission. Mol. Biol. Cell 24, 2319–2327 (2013). 23761076 10.1091/mbc.E12-11-0785 PMC3727925

[6] G. Tarrason Risa ., The proteasome controls ESCRT-III-mediated cell division in an Archaeon. Science 369, eaaz2532 (2020). 32764038 10.1126/science.aaz2532 PMC7116001

[7] A. Booth, C. J. Marklew, B. Ciani, P. A. Beales, In vitro membrane remodeling by ESCRT is regulated by negative feedback from membrane tension. iScience 15, 173–184 (2019). 31060000 10.1016/j.isci.2019.04.021 PMC6503128

[8] Y. Elani ., Constructing vesicle-based artificial cells with embedded living cells as organelle-like modules. Sci. Rep. 8, 4564 (2018). 29540757 10.1038/s41598-018-22263-3 PMC5852042

[9] S. Deshpande, C. Dekker, On-chip microfluidic production of cell-sized liposomes. Nat. Protoc. 13, 856–874 (2018). 29599442 10.1038/nprot.2017.160

[10] L. Harker-Kirschneck, B. Baum, A. E. Šarić, Changes in ESCRT-III filament geometry drive membrane remodelling and fission in silico. BMC Biol. 17, 82 (2019). 31640700 10.1186/s12915-019-0700-2 PMC6806514

[11] H. Yuan, C. Huang, J. Li, G. Lykotrafitis, S. Zhang, One-particle-thick, solvent-free, coarse-grained model for biological and biomimetic fluid membranes. Phys. Rev. E Stat. Nonlin. Soft Matter Phys. 82, 011905 (2010). 20866646 10.1103/PhysRevE.82.011905

[12] J. Huang, J. Liu, B. Kroll, K. Bertoldi, D. R. Clarke, Spontaneous and deterministic three-dimensional curling of pre-strained elastomeric bi-strips. Soft Matter 8, 6291–6300 (2012).

[13] J. Liu, J. Huang, T. Su, K. Bertoldi, D. R. Clarke, Structural transition from helices to hemihelices. PLoS One 9, e93183 (2014). 24759785 10.1371/journal.pone.0093183 PMC3997338

[14] S. Liu, Z. Yao, K. Chiou, S. I. Stupp, M. Olvera de la Cruz, Emergent perversions in the buckling of heterogeneous elastic strips. Proc. Natl. Acad. Sci. U.S.A. 113, 7100–7105 (2016). 27303040 10.1073/pnas.1605621113 PMC4932972

[15] P. Pieranski, J. Baranska, A. Skjeltorp, Tendril perversion—A physical implication of the topological conservation law. Eur. J. Phys. 25, 613 (2004).

[16] T. Savin ., On the growth and form of the gut. Nature 476, 57–62 (2011). 21814276 10.1038/nature10277 PMC3335276

[17] P. E. Silva ., Perversions with a twist. Sci. Rep. 6, 23413 (2016). 27025549 10.1038/srep23413 PMC4812244

[18] P. E. S. Silva, F. Vistulo de Abreu, M. H. Godinho, Shaping helical electrospun filaments: A review. Soft Matter 13, 6678–6688 (2017). 28858364 10.1039/c7sm01280b

[19] C. Prior, J. Moussou, B. Chakrabarti, O. E. Jensen, A. Juel, Ribbon curling via stress relaxation in thin polymer films. Proc. Natl. Acad. Sci. U.S.A. 113, 1719–1724 (2016). 26831118 10.1073/pnas.1514626113 PMC4763768

[20] L. Chu ., One-dimensional spatial patterning along mitotic chromosomes: A mechanical basis for macroscopic morphogenesis. Proc. Natl. Acad. Sci. U.S.A. 117, 26749–26755 (2020). 33051295 10.1073/pnas.2013709117 PMC7604413

[21] J. Moser von Filseck ., Anisotropic ESCRT-III architecture governs helical membrane tube formation. Nat. Commun. 11, 1516 (2020). 32471995 10.1038/s41467-020-15327-4 PMC7260168

[22] W. M. Henne, N. J. Buchkovich, Y. Zhao, S. D. Emr, The endosomal sorting complex ESCRT-II mediates the assembly and architecture of ESCRT-III helices. Cell 151, 356–371 (2012). 23063125 10.1016/j.cell.2012.08.039

[23] E. D. Goley, N. A. Dye, J. N. Werner, Z. Gitai, L. Shapiro, Imaging-based identification of a critical regulator of FtsZ protofilament curvature in Caulobacter. Mol. Cell 39, 975–987 (2010). 20864042 10.1016/j.molcel.2010.08.027 PMC2945607

[24] C. Vanhille-Campos, A. Šarić, Modelling the dynamics of vesicle reshaping and scission under osmotic shocks. Soft Matter 17, 3798–3806 (2021). 33629089 10.1039/d0sm02012e

[25] J. Schöneberg ., ATP-dependent force generation and membrane scission by ESCRT-III and Vps4. Science 362, 1423–1428 (2018). 30573630 10.1126/science.aat1839 PMC6309985

[26] A. K. Pfitzner ., An ESCRT-III polymerization sequence drives membrane deformation and fission. Cell 182, 1140–1155.e18 (2020). 32814015 10.1016/j.cell.2020.07.021 PMC7479521

[27] H. Turlier, B. Audoly, J. Prost, J. F. Joanny, Furrow constriction in animal cell cytokinesis. Biophys. J. 106, 114–123 (2014). 24411243 10.1016/j.bpj.2013.11.014 PMC3907238

[28] A. A. Pulschen ., Live imaging of a hyperthermophilic archaeon reveals distinct roles for two ESCRT-III homologs in ensuring a robust and symmetric division. Curr. Biol. 30, 2852–2859.e4 (2020). 32502411 10.1016/j.cub.2020.05.021 PMC7372223

[29] C. P. Descovich ., Cross-linkers both drive and brake cytoskeletal remodeling and furrowing in cytokinesis. Mol. Biol. Cell 29, 622–631 (2018). 29282285 10.1091/mbc.E17-06-0392 PMC6004588

[30] R. N. Khaliullin ., A positive-feedback-based mechanism for constriction rate acceleration during cytokinesis in *Caenorhabditis elegans*. eLife 7, e36073 (2018). 29963981 10.7554/eLife.36073 PMC6063732

[31] A. Paraschiv ., Influence of membrane-cortex linkers on the extrusion of membrane tubes. Biophys. J. 120, 598–606 (2021). 33460596 10.1016/j.bpj.2020.12.028 PMC7896025

[32] N. Chernov, *Circular and Linear Regression: Fitting Circles and Lines by Least Squares* (Chapman & Hall/CRC Monographs on Statistics & Applied Probability, Taylor & Francis, 2010).

[33] M. Sadeghi, F. Noé, Large-scale simulation of biomembranes incorporating realistic kinetics into coarse-grained models. Nat. Commun. 11, 2951 (2020). 32528158 10.1038/s41467-020-16424-0 PMC7289815

