# Supplementary material for: Physical mechanisms of ESCRT-III–driven cell division
Source: Proc Natl Acad Sci U S A. 2022 Jan 4;119(1):e2107763119. doi: 10.1073/pnas.2107763119 (PMC8740586; doi:10.1073/pnas.2107763119)
Supplement: Supplementary File [file pnas.2107763119.sapp.pdf]

Supplementary Information for

Physical mechanisms of ESCRT-III-driven cell division

Lena Harker-Kirschneck, Anne E. Hafner, Tina Yao, Christian Vanhille-Campos, Xiuyun Jiang, Andre Pulschen, Fredrik Hurtig, Dawid Hryniuk, Siân Culley, Ricardo Henriques, Buzz Baum and Anđela Šarić

Corresponding Author: Anđela Šarić.
E-mail: a.saric@ucl.ac.uk

This PDF file includes:

Supplementary text
Figs. S1 to S13 (not allowed for Brief Reports)
Legends for Movies S1 to S4
SI References

Other supplementary materials for this manuscript include the following:

Movies S1 to S4

Supporting Information Text

This section contains: (1) details of the simulation setup, (2) additional simulations studying the relationship between the rate/protocol of the filament constriction and the filament geometry, (3) the dependence of the midcell diameter evolution on the rate of filament disassembly for instantaneous constriction, (4) additional simulations examining the role of filament curvature change in the sequential and randomised protocol, (5) the division evenness achieved for the sequential and randomised protocol, (6) additional simulations studying the direct influence of filament tension on division success, (7) the thermodynamic contributions in the sequential and randomised protocol, (8) additional simulations and measurements for cells filled with cytoplasmic particles, (9) the repetition of our main results (from simulations) with a triangulated membrane model, (10) the method of measuring the midcell diameter in experiments (11) the method of rescaling the midcell diameter evolution, (12) examples of hemihelices observed experimentally in yeast ESCRT-III filaments.

1. Simulation setup

The cell membrane is modelled using the coarse-grained, solvent free, one-particle-thick membrane model by Yuan et al. (1). Following the original paper, the membrane beads have a diameter σ, where σ is also the MD unit of length, and the inter-bead interaction parameters are set to: ϵ_{memb}=4.34 $\text{k}_\text{B}T$, ξ=4, μ=3, and r_{cut}=1.12 σ. These parameters reproduce a fluid deformable membrane with a bending rigidity of 15 $\text{k}_\text{B}T$, which is in the physiological regime. The membrane includes 48002 particles and forms a vesicle of radius $R_{\text{cell}} = 52.464\ \sigma$.

The ESCRT-III filament in the model consists of three beaded subunits, each of diameter σ, which form rigid bodies (see inset in Fig.1b). The beads of neighbouring subunits are connected by nine harmonic bonds, whose spring constant is set to $600k_BT$. The bond lengths are set to result in an intrinsic filament curvature and the filament curvature can be adjusted by varying the bond lengths. This curvature is the same throughout the filament, hence the relaxed state of the filament is a ring of radius R_{target}. Since the filament subunits cannot overlap with each other, if the filament consists of more than one turn, the resulting relaxed geometry will be a helix instead of a ring.

The blue beads in the filament interact with membrane beads that are at distance r_{ij} via a cut-and-shifted Lennard-Jones potential: $E_{ij} = 4\epsilon\left((\sigma/r_{ij})^{12} - (\sigma/r_{ij})^{6}\right) - E_c,\ r_{ij} < r_c$, with ϵ=4 $\text{k}_\text{B}T$ and the cut-off-distance r_c=1.46 σ. $E_c = 4\epsilon\left((\sigma/r_c)^{12} - (\sigma/r_c)^{6}\right)$ guarantees the continuity at the cut-off-distance. This value of filament-membrane interaction was chosen such that it is within a physiological regime of non-covalent interactions: strong enough to keep the filament on the membrane, but also allowing for filament detachment and filament relaxation upon geometry changes. Fig. S1 shows that the qualitative filament behaviour does not change with the exact choice of this interaction parameter, as long as it is in the physiological regime. The precise region of productive division shown in Fig. 2 merely shifts or broadens slightly. In contrast, the red beads of the filament and the membrane particles, as well as the beads of the filament with each other, only interact via volume exclusion, implemented via a Lennard-Jones potential of ϵ=2 $\text{k}_\text{B}T$, cut and shifted at the minimum of the potential.

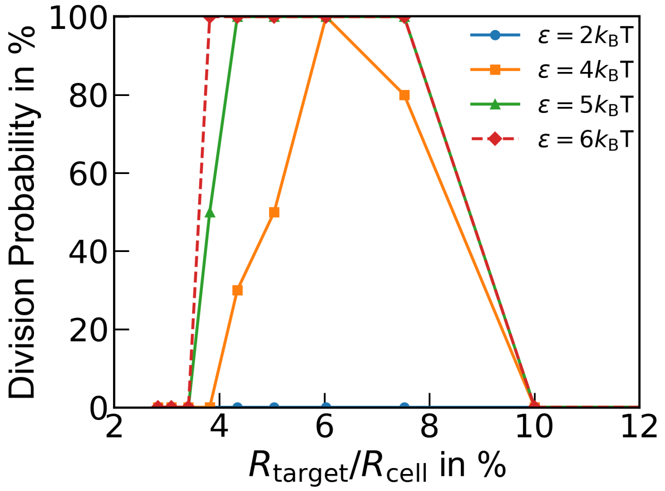

Fig. S1. Repeating the main results from Fig. 2 for different choices of filament-membrane interaction ϵ, keeping $10^2 v_{dis} = 2.7/\tau$ constant in an instantaneous protocol. For $\epsilon = 2k_BT$ the filament does not attach at all, for $\epsilon = 4 - 6k_BT$ the division probability depends non-monotonically on the filament curvature change, showing that the conclusions drawn from Fig. 2 are robust against the choice of the membrane-filament interactions, within the physiologically sound interaction regime.

We perform molecular dynamics (MD) simulations using the molecular dynamics package LAMMPS (2) to integrate the equations of motion with periodic boundary conditions in the canonical ensemble. This was achieved by coupling the $N_{particles}V_{box}E_{system}$ setup ($N_{particles}$ is the total number of particles in the box of a volume V_{box}, and E_{system} is the total energy of the system) to a Langevin thermostat with the friction coefficient set to unity, γ=m/τ, where m is the particle mass (set to unity for all particles) and τ is the MD unit of time. The simulation box is a cube with a fixed edge length of

$L_{\mathrm{box}} = 200\,\sigma$. The MD time-step was chosen to be 0.01τ. At the beginning of every simulation, we first equilibrate the vesicle on its own, and then the filament in contact with the vesicle, where the target radius of the filament equals that of the cell. At the end of this equilibration stage the cells look like in the first snapshots in Fig.1c. The simulation results are visualized using OVITO (3), an open-source analysis and visualization tool. All the scripts and input files necessary to repeat our simulation setup are available on the following link *.

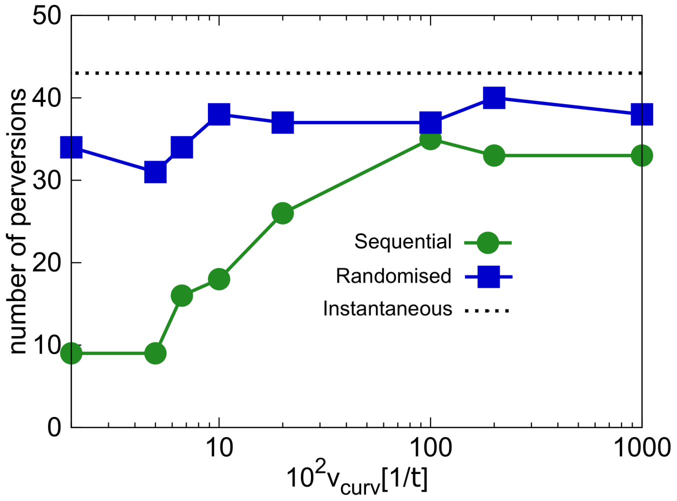

Fig. S2. Filament coiled according to randomised protocol displays more perversions (blue curve) than the one coiled according to the sequential protocol (green curve) with the same rate. Instantaneous protocol displays the largest number of perversions. This data has been collected on a filament attached to a membrane without disassembly, as this scenario is more relevant for the division mechanism than the filament in solution (which displays the same trend). The amount of the curvature change is R_{target}/R_{cell} = 5.0%.

2. Filament geometry depends on the constriction rate and protocol

To understand how the rate of curvature change v_{curv} influences the division probability, we systematically study its influence on the filament geometry. To this end, the simulations for a constriction to 5% of the original cell radius were repeated without disassembling the filament for the instantaneous, sequential, and randomised curvature change protocol. The number of perversions (4, 5) that occurred in the equilibrated filament were measured as a function of v_{curv}. As shown in Fig. S2, for sequential constriction, a faster constriction leads to the formation of more perversions, whereas for randomised constriction the number of perversions generated seems to be rate-independent. Instantaneous constriction generates the most perversions.

The randomised protocol distributes the perversions more evenly along the filament, hence it also leads to a higher constriction of the filament overall. As visible from Fig S3a, the filament's overall radius of gyration is smaller when the filament is constricted to the same degree in the randomised than in the sequential protocol. This higher overall supercoiling of the filament in turn causes higher filament tension and higher membrane constriction (Fig S3b and c). The data in Fig S3 were collected with the disassembling filament to capture the division process.

3. Midcell diameter does not depend on the disassembly rate

Constricting the filament can drive the formation of small bottlenecks, however, disassembly is necessary to achieve robust cell division. The disassembly rate v_{dis} has to be well adjusted to the time it takes for the bottleneck to establish. In Fig. S4, we plot the cell diameter as a function of time for $R_{\mathrm{target}}/R_{\mathrm{cell}} = 5.5\%$ and various disassembly rates v_{dis} following the instantaneous constriction protocol.

We can see that all lines initially follow the same general curve, but if the filament disassembles before it can form a sufficiently small bottleneck, the cell membrane recovers its initial spherical shape and the diameter increases again. For disassembly rates slower than $10^2 v_{\mathrm{dis}} = 6.67\tau$, the bottleneck can be maintained long enough for the cells to divide. If we only look at cells that divide, the disassembly rate has no influence on the shape of the midcell evolution curve. Hence for a given v_{curv}, we can use all the simulations that divided for all different values of v_{dis} when performing statistical analysis and thereby increase our sample size significantly.

* https://github.com/cvanhille/ESCRTIII_CD_ex

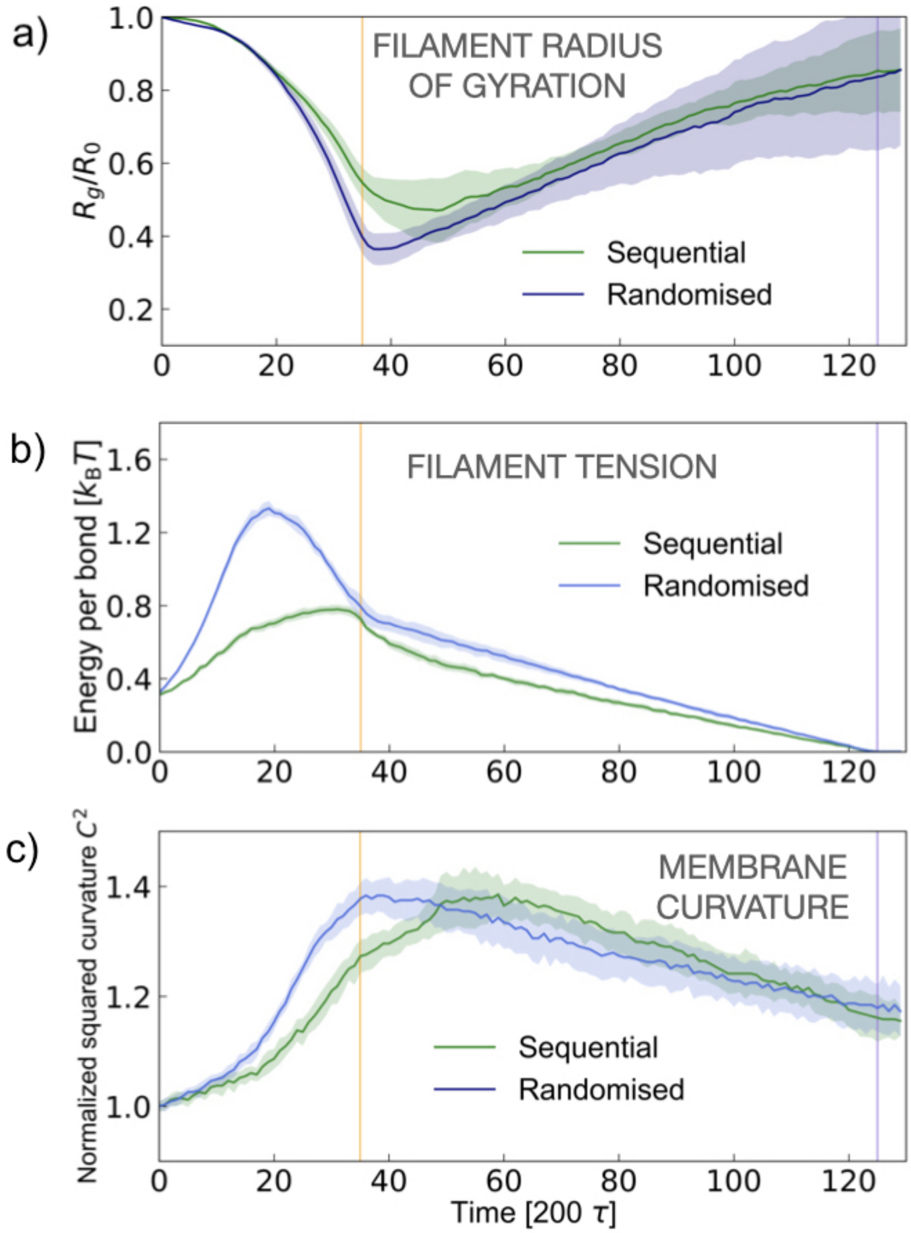

Fig. S3. The filament coiled according to randomised protocol (blue curves) has a smaller radius of gyration (top panel), higher tension (middle panel), and creates higher membrane curvature (bottom panel) than the one coiled according to the sequential protocol (green curves). In all the panels, the membrane was present and the filament was disassembled following the constriction. Orange and purple vertical lines indicate the start and the end of the filament disassembly, respectively. Speed of constriction: $10^2 v_{curv} = 6.7/\tau$; speed of disassembly: $10^2 v_{dis} = 2.7/\tau$; and curvature reduction: $R_{target}/R_{cell} = 5.5\%$.

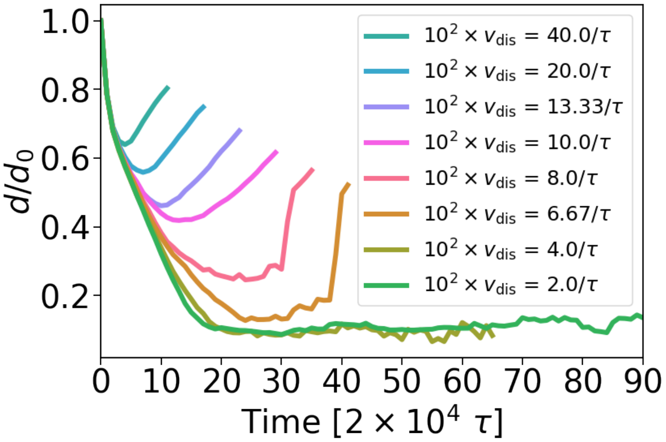

Fig. S4. Midcell diameter evolution after constricting the filament instantaneously by $R_{\rm target}/R_{\rm cell} = 5.5\%$ and varying the disassembly rate $v_{\rm dis}$. Each curve is the mean of 10 random seeds. Note that disassembly must be slow ($10^2 v_{\rm dis} < 6.67\tau$) for divisions to complete.

4. Varying filament constriction in the sequential and randomised protocol

In the manuscript we measure how the division probability and evenness depend on how much we change the filament curvature R_{target}/R_{cell} for the instantaneous protocol (Fig.2, Fig.3). These measurements explore the role of the force that the filament exerts — showing that division fails if this force is too large or too small. To test if these conclusions depend on the exact protocol of force application, we have repeated the same measurements for the sequential and randomised protocol. As shown in Fig. S5 (continuous lines), division fails for too high or too low filament curvature changes. Interestingly, the region for the successful division is broader for the randomised curvature change. Please note that, because of this, to see the full non-monotonic behaviour for the randomised curvature we would need to go to very low R_{target} values, which is prohibitive in our model due to filament volume exclusion.

Cells appear to divide less evenly when using the sequential protocol than the randomised or instantaneous protocol, which both achieve similar amounts of division evenness (dashed lines). However, the randomised protocol achieves this high symmetry of division for a broader range of curvature changes.

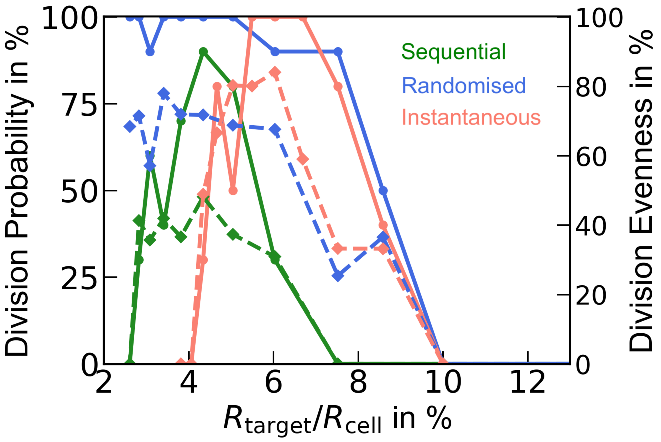

Fig. S5. The division probability (continuous lines) vs the amount of the curvature change for the sequential and randomised curvature change, showing the same non-monotonic behaviour as the instantaneous protocol in Fig. 2. The dashed lines compare the division evenness between the three protocols. The rate of curvature change was set to the value that matched best with the normalized cell diameter evolution curve (Fig.6): $10^2 v_{curv} = 5/\tau$ for the sequential and $10^2 v_{curv} = 16/\tau$ for the randomized protocol; the rate of disassembly is $10^2 v_{dis} = 2.7/\tau$.

5. Division evenness for the sequential and randomised protocol

Fig. S6 shows how evenly the cells divide on average (using 10 different seeds per square), depending on the disassembly rate $v_{\rm dis}$ and the rate at which the filament contracts $v_{\rm curv}$ for non-instantaneous constriction protocols. Division symmetry is measured by assessing how much of the ESCRT-III polymer ends up in the two daughter cells. For the sequential protocol division is generally not very even, with even division occurring only for fast constriction rates (Fig. S6a). The randomised protocol yields very even division, regardless of the constriction or disassembly rate (Fig. S6b).

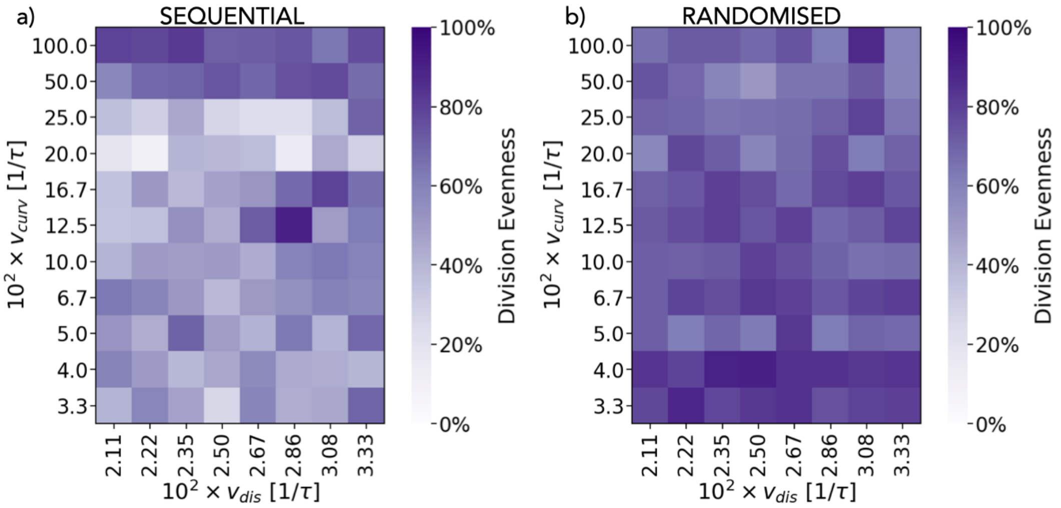

Fig. S6. a) The influence of the constriction rate v_{curv} and the rate at which the filament is disassembled v_{dis} on how evenly the cells divide following the sequential protocol. The colour represents the division evenness E, as defined in the main text. If $E = 100\%$ the daughter cells contain the exact same amount of filament subunits. Each square shows the average Evenness E out of 10 simulations performed with different seeds. b) The same as in a) but following the randomised regime. Here the cells all divide very evenly regardless of the parameters we choose.

6. The role of filament tension in division

For simplicity, in the manuscript we kept the filament bond strength at $k = 600k_BT$. Here we varied the filament bond strength to probe its effect on division probability when using the randomised protocol. As shown in Fig. S7, our simulations predict that if the tension in the filament is decreased, division becomes less reliable. In experiments this can be tested by deleting one of the two proteins that make up the contractile CdvB1/2 filament. Deleting either one of the proteins would yield a filament that has lower tension. Indeed, in experiments we find that the deletion of either B1 or B2 proteins produces less reliable or slower division (6), in agreement with the predictions of our model.

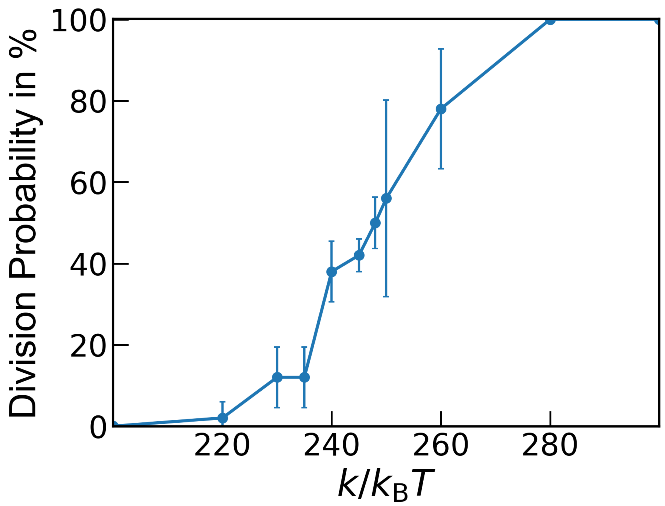

Fig. S7. The average division probability vs the amount of tension in the filament, showing that below a certain tension threshold division fails. The simulations were carried out using the randomised curvature change protocol with a rate of curvature change: $10^2 v_{curv} = 16/\tau$; rate of disassembly: $10^2 v_{dis} = 2.7/\tau$; and curvature reduction: $R_{target}/R_{cell} = 5.0\%$.

7. Thermodynamic work for different curvature change protocols

The sudden switch of the filament target radius from R_{cell} to R_{target} leads to a surge of energy in the filament bonds, which is able to perform work. The theoretical limit of the energy input in such a non-equilibrium process equals to $E_{\text{input}} = (N_{\text{mol}} - 1) \cdot \sum_{n=1}^{9} k \cdot (l_n^f - l_n^i)^2$, where N_{mol} is the number of filament subunits, k is the spring constant of the harmonic bonds, l_n^i and l_n^f are bond lengths for the nth bond between any pair of neighbouring subunits f and i associated with the initial and final target radius R_{cell} and R_{target}, respectively.

The difference in energies before and after the constriction process was computed and decomposed into four contributions:

- Decrease in filament-membrane binding interactions $E_{\text{detachment}}$. Initially, the filament is fully attached to the membrane, but becomes partially detached after the constriction, which leads to an energy increase.
- Increase in membrane energy $\Delta E_{\text{membrane}}$ due to an increase in curvature, which is computed according to the membrane model used in the simulation (1).
- Increase in filament bond energy $\Delta E_{\text{filament}}$, due to perversions and supercoiling.
- Dissipation is computed as
  $E_{\text{dissipation}} = E_{\text{input}} - E_{\text{detachment}} - \Delta E_{\text{membrane}} - \Delta E_{\text{filament}}$.

We find that consistently around 75% of the supplied energy to the filament is dissipated, but we do not observe a significant difference in dissipation between different protocols (Fig. S8). However, there is a substantial difference in how the non-dissipated portion of the energy is spent in different protocols. In the randomised curvature change protocol the highest proportion of the non-dissipated energy supplied to the filament is transferred into membrane deformation, which is the productive work in our case, while in the sequential protocol the highest proportion is transferred into filament detachment. This is consistent with the resulting higher fidelity of division in the randomised protocol measured in our simulations.

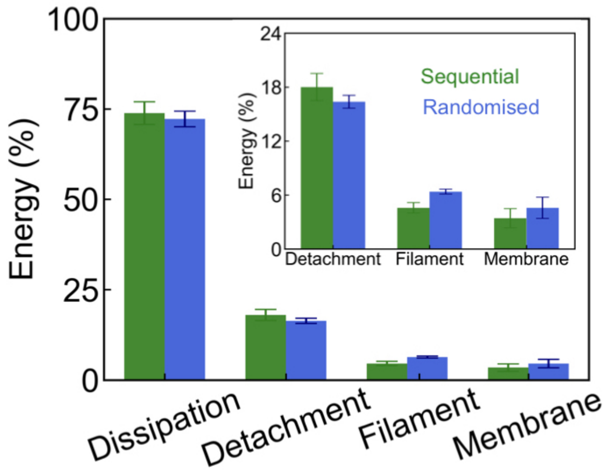

Fig. S8. The average partitioning of the invested energy in the sequential and randomised protocol when constricting by the same amount and using the same constriction and disassembly rate. On average $\sim 75\%$ of the energy invested in the curvature change is dissipated. In the randomised protocol more of the non-dissipated potion of energy is spent on productive work (membrane deformation) than in the sequential protocol. All the energies were computed directly from the relative potential energies by taking the difference between the final and initial energies. Rate of curvature change: $10^2 v_{curv} = 20/\tau$; rate of disassembly: $10^2 v_{dis} = 2.7/\tau$; and curvature change $R_{target}/R_{cell} = 5.5\%$.

8. The effect of cytoplasmic volume

The simulation setup in the main paper does not account for the fact that cells are filled with cytoplasmic content. 20-30% of a cell's volume is occupied by proteins (7). In order to investigate the presence of non-compressible cytoplasmic content on the dynamics of cell division, we place volume-excluded particles inside the simulated cells. The volume exclusion is implemented via a Lennard-Jones potential of ϵ=2 $k_B T$, cut and shifted at the potential minimum.

The hexagonal close packing arrangement is used to place the cytoplasmic particles inside the cells at high packing fraction, as shown in Fig. S9a. We then explore the influence of the packing fraction (the percentage of volume inside the cell taken up with cytoplasmic particles), as well as the cytoplasmic particle diameter σ_{cyto}, on cell division. Simulations are run for ten different seeds at packing fractions 10-40%, $\sigma_{cyto} = 5, 6, 7, 8\sigma$ and for the three different curvature change protocols. We fix the amount by which we reduce the target radius of the filament to $R_{\text{target}}/R_{\text{cell}} = 6\%$ and the rate at which the filament disassembles to $10^2 v_{\text{dis}} = 3.3/\tau$. The division probability depending on the packing fraction and the cytoplasmic particle diameter is represented via the colour of each square in Fig. S9c. The number of cytoplasmic particles in each simulation is also included in the diagram.

Fig. S9c shows that an increasing number of cytoplasmic particles lowers the likelihood of successful division. This result is to be expected as the presence of particles within the simulated cell causes internal pressure that acts against the constricting force of the filament protein that drives cell division. Fig. S9d shows the time evolution of the average midcell diameter (over 10 seeds) for varying packing fractions using the randomised protocol. For all packing fractions, the filament was constricted to 6% of its original cell radius and the cytoplasmic particle radius was fixed at $\sigma_{cyto} = 8\sigma$, because it is the configuration with the greatest amount of variation in cell division success.

The greater the packing of the cell, the slower the rate of constriction. At very high packing fractions division fails. Importantly, if division succeeds (for $0-25\%$ packing), the midcell evolution curve always follows the same general shape. Hence, the difference between the experimental and simulated curve in Fig.6d likely cannot be explained by the lack of the resistance of the cytoplasmic volume in the initial model.

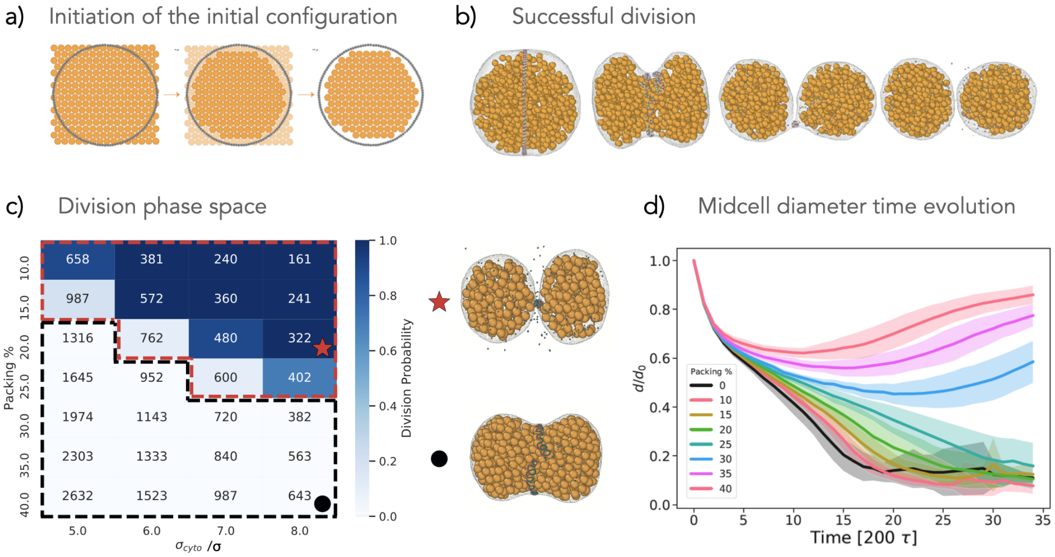

Fig. S9. Investigating the role of the cytoplasmic volume in the division process. a) The protocol of how cytoplasmic particles are initialised in the cell model. b) A representative example of a successful division. Here the diameter of the cytoplasmic particle is $\sigma_{cyto} = 8\sigma$ and the packing fraction is 20%. c) The probability of successful cell division depending on the packing fraction and the diameter of the cytoplasmic particles, σ_{cyto}. The number inside each cell represents the number of cytoplasmic particles in the cell. d) The midcell diameter evolution for different packing fractions with $\sigma_{cyto} = 8\sigma$.

9. Triangulated membrane

For the purpose of showing that the ESCRT-III constriction mechanism described in this paper is independent of the particular choice of membrane model, we repeated our main results using a coarse-grained tiangulated membrane model governed by a Helfrich Hamiltonian (which considers both bending and stretching contributions) within a Monte Carlo scheme.

The system we consider here thus consists of two elements: i) a vesicle modelled as a triangulated thin elastic sheet with hard sphere beads situated at every vertex representing the membrane lipid patches, as in (8) and ii) a filament of three-beaded monomers like the ones described in the main text, connected by harmonic bonds which control its target geometry. The dynamics of the system are evolved through a Monte Carlo scheme that involves four types of moves: vertex displacement moves, membrane bond flip moves, filament monomer displacement moves and filament monomer rotation moves. The vertex displacement moves mimic the lateral diffusion of lipids, whilst also allowing for vertical membrane fluctuations. The membrane bond flip moves ensure that the membrane preserves its fluidity by dynamically rearranging the connectivity. Finally, the monomer displacement and rotation moves allow for the relaxation of the filament to its target geometry. The membrane energy is described using Helfrich Hamiltonian (8), and we chose parameters encoding for a bending rigidity of $\kappa = 15\ k_B T$, which is in the range of physiologically relevant lipid membranes and also matches our membrane model used in the main text. We have also set the bond constant $K_{\text{fil}} = 2000\ k_B T$ to guarantee sufficient filament stiffness, and we have set the interaction constant $\epsilon = 3\ k_B T$.

This membrane model cannot reproduce scission, but can reproduce substantial constriction of the midcell diameter. For the instantaneous filament curvature change we observe qualitatively analogous behaviour to our molecular dynamics results for the success of division – membrane remodelling fails for too little/too much tension, but is successful for an intermediate regime. Also the midcell diameter evolution in time (the exponential curve shape) qualitatively resembles the one observed in our molecular dynamics simulations with a different membrane model (see Fig.S10). This reinforces our confidence in the notion that our results are not model dependent. It is however important to add that the dynamics of the filament becomes very slow within the deformed cell neck in Monte Carlo, and to recover full constriction to a tight neck, global filament Monte Carlo moves would be required, as is common in Monte Carlo simulations of polymers at high local densities. We have decided not to do this here, as it would also not be able to provide us with dynamical information.

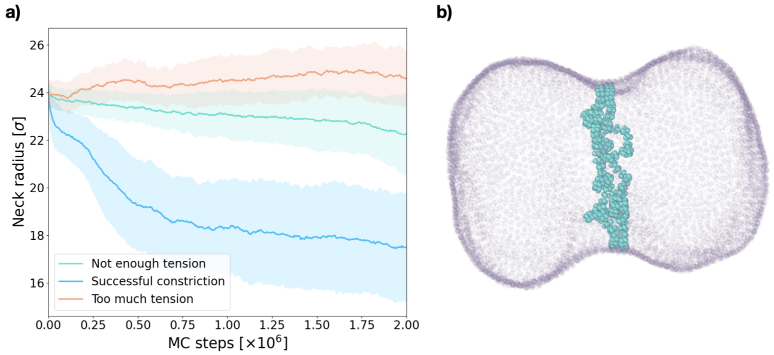

Fig. S10. Monte Carlo simulation of a triangulated membrane. a) Midcell radius evolution over time (Monte Carlo steps) for three representative scenarios. Green curve - *Not enough tension*: The filament target curvature radius is around 33% of the initial cell radius, and it does not have enough tension to deform the membrane. Blue curve - *Successul constriction*: With target curvature radius around 9.5% of the initial one, the filament here has the right amount of tension to drive considerable neck constriction. Orange curve - *Too much tension*: For target curvature radius around 7.5% of the initial one, the filament has too much tension and detaches from the membrane. b) Snapshot of the constriction scenario after relaxation. It is interesting to note the numerous perversions formed along the filament, also observed in the molecular dynamics simulations (see main text).

10. Measurements of the midcell diameter

The microscopy field of view covered an area of $\sim 150\mu m \times 150\mu m$, displaying multiple cells at once. We used a machine learning algorithm to detect any dividing cells and cropped them from the master image stack (using ImageJ) to a more appropriate scale as can be seen in Fig. S11.

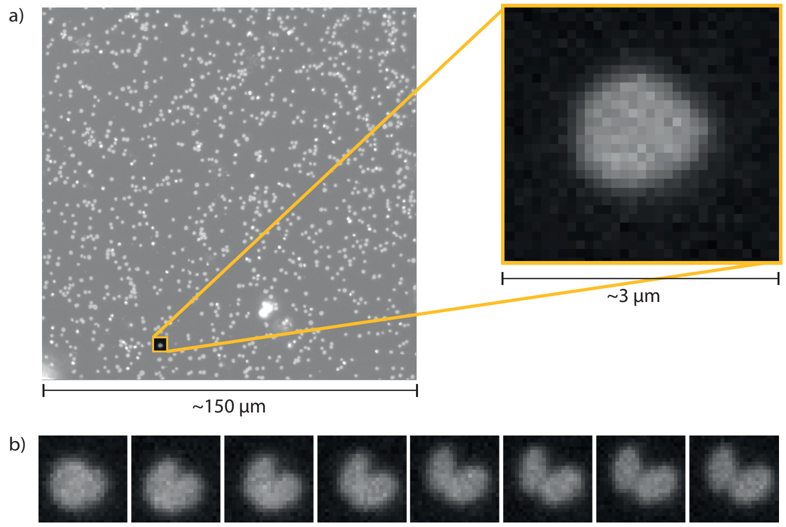

Fig. S11. a) A dividing cell cropped out of a time-lapse film of MW001 *S. acidocaldarius* cells using ImageJ software. b) A montage of images showing how the cropped cell divides over time, with one image taken every minute.

The resolution of movies of dividing *S. acidocaldarius* were limited by the small size of the cells and the resolution of the microscope. The Full-Width Half-Maximum (FWHM) of the point-spread function of the microscope for this experiment was estimated to 632 nm. It is therefore not straightforward to determine the edges of the cell. The FWHM of an intensity profile can be used to describe the measurement of the width of an imaged object when the edges of the image are not sharp (9). The intensity profile of the midcell diameter can be extracted using ImageJ's line tool as shown in Fig. S12a.

We then fitted the intensity profiles via a Gaussian, and calculated the FWHM of the fit via

$$FWHM = 2\sqrt{2ln2}\sigma_G \approx 2.355\sigma_G,$$

where σ_G is the standard deviation of the fitted Gaussian.

Some images of the cells have two intensity peaks corresponding to two Gaussians. The two Gaussians are present in the early stages of cell division, as shown in the left panel of Fig. S12b. This is due to the overlapping membranes of the daughter cells that create two localised areas of increased intensity. As the cell divides, the two areas move closer together until the two intensity peaks form one.

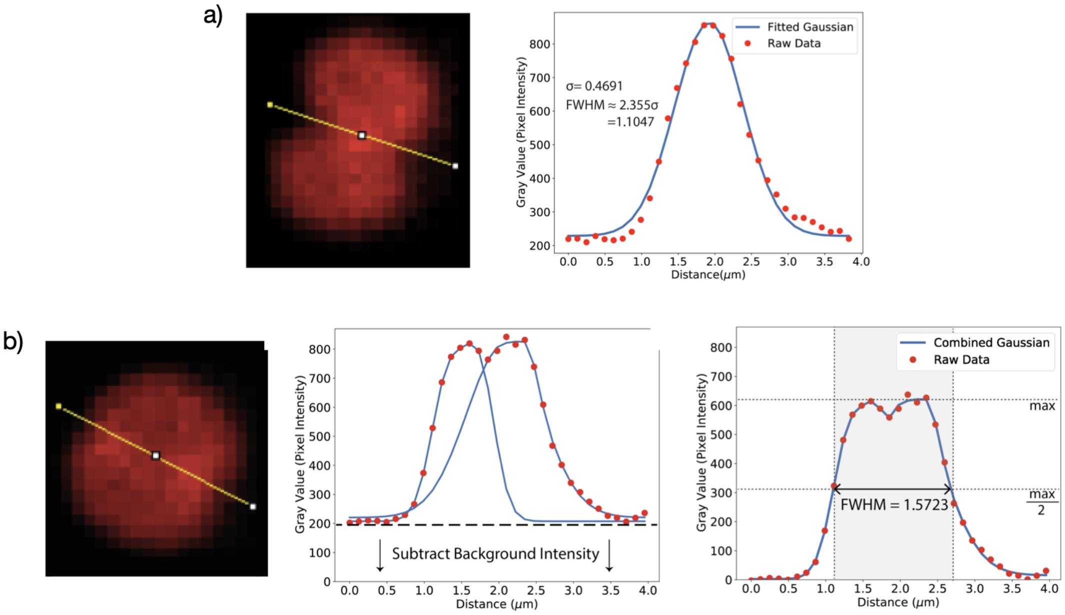

Fig. S12. a) Left panel: The intensity over the midcell axis of a cell is measured using the line tool in ImageJ. Right panel: The intensity is plotted against distance and fitted with a Gaussian curve. The FWHM of the Gaussian is calculated by multiplying the standard deviation of the curve by $2\sqrt{2ln2}$. b) In the early stages of division, cells appear to have two intensity peaks on the midaxis of the cell due to the overlapping of daughter cells. Left panel: The intensity over the midcell axis of a cell is measured using the line tool in ImageJ. Middle panel: The intensity is plotted against distance and fitted with two Gaussian curves, then zeroed by subtracting the background intensity. Right panel: The two Gaussians are then joined, and the FWHM is calculated from the combined Gaussian.

If two peaks were present, two Gaussians were fitted to the profile instead of one. These fits were background-subtracted such that their offsets were zero (Fig. S12b, middle panel), and then combined to create a unified curve (Fig. S12b, right panel). This process was repeated for each frame of the data where the profile was poorly represented by a single Gaussian. The combined double-Gaussian fit does not have a single well-defined σ_G. In this case, the FWHM was measured as the width of the combined fit profile at half the intensity of the higher-amplitude peak.

11. Midcell diameter evolution rescaling

The shape of the curves describing the midcell diameter of the dividing cells over time resemble a sigmoidal function, but can be quite asymmetric if the filament curvature changes quickly. We hence fitted them using this generalised logistic function that allows the curves to be both asymmetric and symmetric:

$$Y(t) = A + \frac{K - A}{(1 + e^{-B(t-M)})^{1/\nu}},$$

with A being the lower and K the upper asymptote. B is the growth rate and ν determines how asymmetric the curve gets. M can be considered the starting time at which the curve turns: $Y(t = M) = A + \frac{K - A}{2^{1/\nu}}$. To improve the fit quality, we chose $\nu = 1$ for the experimental fits, as they are very symmetric. For the simulated curves, we chose the upper asymptote $K = 1$, since we know they all start at the same original diameter.

We then fit the diameter over time (for all random seeds) using this function and extract the fitting parameters to scale the curve in the x and y direction. Scaling in the y-direction is simple, as we only need to subtract the minimum asymptote value from the data points and then divide them by the difference between the upper and lower asymptote: $\text{Diameter}_{\text{scaled}} = (\text{Diameter} - A)/(K - A)$. Now the y-axis is given in percentage of original diameter, rather than the diameter in μm. Next we scale the x-axis from time in min to percentage of completed division. To do this we measure the time points at which the fits reach 99% (T_{start}) and 1% (T_{end}) of their maximal values respectively. We then subtract the starting time from all x-values and divide by the difference between the starting and ending time, which is the amount of time the cell took to divide: $\text{Time}_{\text{scaled}} = (\text{Time} - T_{\text{start}})/(T_{\text{start}} - T_{\text{end}})$. Once scaled, we interpolate the midcell diameter evolution curves for all simulation seeds and calculate their mean and standard deviation, which is then displayed in Fig. 6d-f.

12. Hemihelices in ESCRT-III filaments

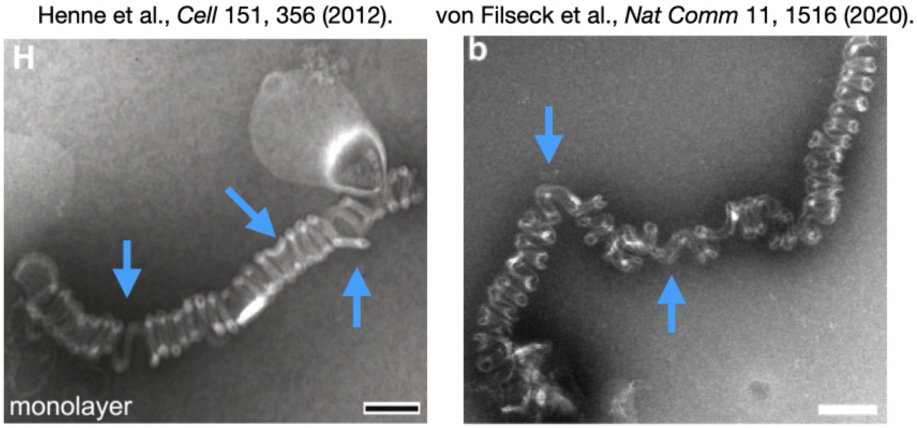

Fig. S13. Examples of hemihelices reported in eukaryotic (yeast) ESCRT-III filaments. Left panel: Snf7^{R52E}/Vps24/Vps2 helices assembled on lipid monolayers, reprinted with permission from Henne et al. (10). Examples of perversions are marked with blue arrows and the scale bar is 100nm. Right panel: Snf7/Vps24/Vps2 filaments grown on large unilamellar vesicles. Reprinted with permission from von Filseck et al. (11) under Creative Commons Licence https://creativecommons.org/licenses/by/4.0/ with minor changes (blue arrows added). Examples of perversions are marked with blue arrows and the scale bar is 100nm.

Movie S1. Example of a successful cell division simulation following the instantaneous constriction protocol. Here $R_{\text{target}}/R_{\text{cell}} = 5\%$, $10^2 \times v_{\text{dis}} = 6.7/\tau$.

Movie S2. Example of a successful cell division simulation following the sequential constriction protocol for a fast curvature change rate, $10^2 \times v_{\text{curv}} = 100.00/\tau$. $R_{\text{target}}/R_{\text{cell}} = 5\%$ and $10^2 \times v_{\text{dis}} = 2.11/\tau$.

Movie S3. Example of a failed cell division simulation following the sequential constriction protocol performed at a slow rate, $10^2 \times v_{\text{curv}} = 20.00/\tau$. $R_{\text{target}}/R_{\text{cell}} = 5\%$ and $10^2 \times v_{\text{dis}} = 2.11/\tau$.

Movie S4. Example of a successful cell division following the randomised constriction protocol at a moderate rate, $10^2 \times v_{\text{curv}} = 5.00/\tau$. $R_{\text{target}}/R_{\text{cell}} = 5\%$, $10^2 \times v_{\text{dis}} = 3.08/\tau$.

References

1. H Yuan, C Huang, J Li, G Lykotrafitis, S Zhang, One-particle-thick, solvent-free, coarse-grained model for biological and biomimetic fluid membranes. *Phys. Rev. E* **82**, 011905 (2010).
2. S Plimpton, A Thompson, S Moore, A Kohlmeyer, Lammps documentation (2017).
3. A Stukowski, Visualization and analysis of atomistic simulation data with ovito — the open visualization tool. *Model. Simul. Mater. Sci. Eng.* **18**, 015012 (2010).
4. J Huang, J Liu, B Kroll, K Bertoldi, DR Clarke, Spontaneous and deterministic three-dimensional curling of pre-strained elastomeric bi-strips. *Soft Matter* **8**, 6291–6300 (2012).
5. S Liu, Z Yao, K Chiou, SI Stupp, MO De La Cruz, Emergent perversions in the buckling of heterogeneous elastic strips. *Proc. Natl. Acad. Sci.* **113**, 7100–7105 (2016).
6. AA Pulschen, et al., Live cell imaging of the hyperthermophilic archaeon sulfolobus acidocaldarius identifies complementary roles for two escrtiii homologues in ensuring a robust and symmetric cell division. *bioRxiv* (2020).
7. R Ellis, Macromolecular crowding: obvious but underappreciated. *Trends Biochem. Sci.* **26**, 597 – 604 (2001).
8. A Paraschiv, et al., Influence of membrane-cortex linkers on the extrusion of membrane tubes. *Biophys. J.* **120**, 598–606 (2021).
9. F Zhao, *Confocal Microscopy Tutorial: Lateral And Axial Resolution In Confocal System.*, (2004 (Accessed 6 March 2020)) http://www.hi.helsinki.fi/amu/AMU%20Cf_tut/cf_tut_part1-5.htm.
10. WM Henne, NJ Buchkovich, Y Zhao, SD Emr, The endosomal sorting complex escrt-ii mediates the assembly and architecture of escrt-iii helices. *Cell* **151**, 356–371 (2012).
11. JM Von Filseck, et al., Anisotropic escrt-iii architecture governs helical membrane tube formation. *Nat. communications* **11**, 1–9 (2020).
